# Quantitative immunohistochemical expression of c Kit in breast carcinomas is predictive of patients' outcome

**DOI:** 10.1038/sj.bjc.6605113

**Published:** 2009-06-09

**Authors:** C Charpin, S Giusiano, S Charfi, V Secq, S Carpentier, L Andrac, M-N Lavaut, C Allasia, P Bonnier, S Garcia

**Affiliations:** 1 Department of Pathology, Hôpital Nord and Université de la Méditerranée (Aix Marseille II), Marseille, France; 2 Department of Gynecologic Oncology, Hôpital de la Conception, Marseille, France

**Keywords:** c Kit, quantitative immunohistochemistry, breast carcinomas

## Abstract

**Background:**

c Kit (CD117) expression in tissues has been reported as a relevant target for specific therapy in some human malignancies, but has been poorly documented in breast carcinomas

**Methods:**

The prognostic significance of c Kit in a series of 924 breast carcinomas (mean follow-up, 79 months) was investigated using standardised high-throughput quantitative densitometry of immunohistochemical precipitates in tissue microarrays.

**Results:**

c Kit was expressed in 14.7% breast carcinomas (and in 42 out of 586 node-negative tumours). In univariate analysis, (log-rank test) the score of c Kit expression correlated with poor patient outcome *P*=0.02 and particularly in node-negative cases (*P*=0.002). In multivariate Cox analysis, c Kit was an indicator of metastasis independent of 25 other concomitantly evaluated markers of prognosis. Logistic regression showed that c Kit ranked 10 out of 25 (*P*=0.041), and was included in a 10-marker signature that allowed 79.2% of the patients to be correctly classified in the metastatic or metastasis-free categories independently of hormone receptors and HER-2 status. Interestingly, c Kit was also a significant predictor of metastasis in node-negative tumours (2 out of 25 ranking, *P*<0.0001) and included in a six-marker signature of prognosis, correctly classifying 88.6% of the patients (*P*<0.0001).

**Conclusion:**

We concluded that, as assessed by quantitative immunohistochemistry, c Kit is an independent prognostic indicator that could also potentially serve as a target for specific therapy in breast carcinomas.

As a transmembrane tyrosine kinase, c Kit plays a physiological role in the development of several cell types including haematopoietic cells, germ cells and melanocytes (Miettinen and Lasota, 2005). In breast tissue, c Kit is found in normal epithelium and non-neoplastic breast lesions, suggesting a role in the maintenance of breast glandular epithelium (Matsuda *et al*, 1993; Miettinen and Lasota, 2005).

Published data show a low prevalence of c Kit expression in breast carcinomas, ranging from 1 to 13% (Tsuura *et al*, 2002; Nielsen *et al*, 2004; Simon *et al*, 2004; Tsuda *et al*, 2005; Reis-Filho and Tutt, 2008), and in one report up to 25% (Tsutsui *et al*, 2006). The variations in expression in previous immunohistochemical studies can be explained by a lack of standardisation of procedures, particularly of quantification of immunostaining.

Therefore, it is clear that, first, expression of c Kit needs to be evaluated with a high-throughput standardised procedure in large series to determine the real prevalence and the clinical relevance of its identification in individual breast carcinomas. Second, correlation of the levels of c Kit expression with patients' outcome will show its actual prognostic value.

In the present immunohistochemical study of 924 invasive breast carcinomas, we investigated c Kit expression within tissue microarrays (TMA), quantified by densitometry on digitised microscopic images using an image analysis device and dedicated software. The expression of c Kit was correlated with (1) patients' outcome (mean follow-up, 79 months) and (2) that of 25 other prognostic markers, with particular attention to node-negative tumours.

## Materials and methods

### Patients

The participants were a consecutive series of 1200 patients with invasive breast carcinomas who were operated on from 1995 to 2002 (mean follow-up, 79 months) in the same department at the Hôpital Conception, Marseille. Surgery was in all cases the first treatment (PB). For this first step of treatment, patient management was handled by the same group of surgeons and by three senior pathologists (CC, SG, LA). Conservative treatment, mastectomy and node resection (complete or sentinel) were applied according to the current European recommendations. Likewise, radiotherapy, chemotherapy and hormone therapy were applied according to criteria currently used at that time.

Analysis of the distribution of the series by age, histological type and grade, and nodal status before TMA construction revealed the usual distribution of breast carcinomas and no bias in tumour selection, as compared with literature data. Owing to the technical difficulties in carrying out immunocytochemical tests on many serial paraffin sections of a TMA to evaluate the 35 different markers (Table 1), complete data for all markers were finally obtained for only 924 patients out of the initial series of 1200.

The 2005 follow-up data in clinical records showed that 181 out of 924 patients had metastatic tumours.

Our study focused mainly on correlation of quantitative immunohistochemical data with patients' outcome. Current histoprognostic criteria on H and E staining were not retained for statistical analysis, mainly to limit the burden of data and also to focus the statistical analysis on continuous variables homogeneously obtained by (numerical) densitometric measurement of immunoprecipitates with the image analysis device.

### Tissue

Tissue samples were all archival material taken from consecutive surgical specimens after formalin fixation and paraffin embedded blocks. Attention was paid to optimal consistent tissue-handling procedures, including fast immersion in buffered formalin in an appropriate container by pathologists or by nurses trained in the procedure. Tumour fragments were large (5 mm) and thick enough (3 mm) to allow further TMA construction. Duration of fixation was 24 h for smaller samples (<5 cm) and 48 h for larger ones, to improve formalin penetration, before specimen dissection at room temperature. After fixation, paraffin pre-embedding and embedding were carried out with currently available automated devices of the same brand.

Paraffin blocks were stored in the same room, in which temperature was maintained at 20°C before TMA construction.

### TMA construction

The procedure for construction of TMAs was as previously described (Garcia *et al*, 2007a, 2007b). Briefly, cores were punched from the selected 1200 paraffin blocks (from 1200 patients), distributed in six new blocks including two cores for each tumour (200 cases per block, a total of 2400 cores) of 0.6-mm diameter. All the new blocks (TMAs) were stored at 4°C, before sections 4-*μ*m thick were prepared for each marker to be examined by immunohistochemistry.

### Immunohistochemistry

Serial tissue sections were prepared and stored at 4°C for 24 h before immunohistochemical processing, as previously reported (Garcia *et al*, 2007a, 2007b). Immunoperoxidase procedure was performed using an automated Ventana Benchmark XT device and Ventana Kits (Ventana, Strasbourg, Illkirch, France).

Markers were detected using commercially documented antibodies (Table 1). Dilutions of antibodies were determined by pre-screening on the usual full 4-μm thick sections before use on TMA sections.

### Image analysis

Automated densitometric measurements of immunoprecipitates in cores were made for each marker antibody in each core individually identified after digitisation and image cropping of the slides, as previously reported (Garcia *et al*, 2007a, 2007b). Briefly, TMA analysis with a SAMBA 2050 automated device (SAMBA Technologies, Meylan/Grenoble and TRIBVN, Chatillon, France) (Charpin *et al*, 1997b, 1998a, 1998b, 2004) was carried out according to the following protocol.

First, an image of the entire slide was built up using low-power magnification ( × 2, pixel dimension 3.7 *μ*m). This image was made up of a mosaic of images acquired along a rectangular grid with contiguous fields. Second, the area of the slide containing the TMA cores was automatically delineated and scanned at higher magnification ( × 20, pixel dimension 7.4 *μ*m). Third, after autofocusing, the images were acquired with an overlap greater than the largest mechanical positioning error. Using the image contents, a matching algorithm determined precisely the relative position of each image with respect to its neighbours. Calculated overlap was removed from images to produce a new set of higher-magnification images, thus covering precisely the cores of interest. A specially developed tool referred to as TMA crop (Plaisir, France) then allowed superposition of the TMA grid onto the reduced image and precise alignment of each node of the grid with the core location within the image. The final step was carried out automatically using the core image contents to ensure pixel precision of the match. From the images acquired with × 20 magnification, a new set of images was next computed, one for each core. For colour analysis of the core images, the SAMBA ‘immuno’ software was applied as previously reported (Charpin *et al*, 1994, 1997a, 1997b, 1998a, 1998b, 1998c, 2004; Garcia *et al*, 2007a, 2007b) in the usual full-tissue sections.

In the present study, we correlated the patients' follow-up parameters with a quantitative score combining the surface stained and the intensity of staining (Garcia *et al*, 2007a, 2007b) computed by the SAMBA ‘immuno’ software. The threshold for positive staining was determined according to a densitometric measurement of c Kit immunostaining in normal tissue run in the same batch as the TMAs.

### Statistical analysis

Immunohistochemical expression of each marker was first correlated with patients' disease-free survival using NCSS (www.ncss.com) and Statistica statistical software (www.statsoft.com).

When significant differences in mean expression were identified in patients with disease and without disease, the prognostic significance was determined by log-rank tests (Kaplan–Meier curves). The appropriate threshold of prognostic significance for a given marker was determined as previously recommended (Altman *et al*, 1994) and described (Charpin *et al*, 1994, 1997a, 1997b, 1998a, 1998b, 1998c, 2004; Garcia *et al*, 2007a, 2007b).

Logistic regression (with ROC curves) was then used to identify the combination of markers with the best sensitivity and specificity indicative of a proteomic signature of poor prognosis.

Finally, unsupervised hierarchical clustering of significant prognostic indicators in the overall series provided qualitative data to be compared with previously reported research results on relationships between these molecules, and on the role played by them in the process of cancer metastasis.

## Results

### c Kit distribution in node-positive (N+) and node-negative (N−) tumours

The screening of spots by image analyser after TMA ‘cropping’ revealed that 135 (14.7%) of all tumours were c Kit-positive, whereas among node-negative tumours, 42 out of 584 (7.2%) and among node-positive tumours 71 out of 340 (21%) were c Kit-positive.

**Figure 1:**
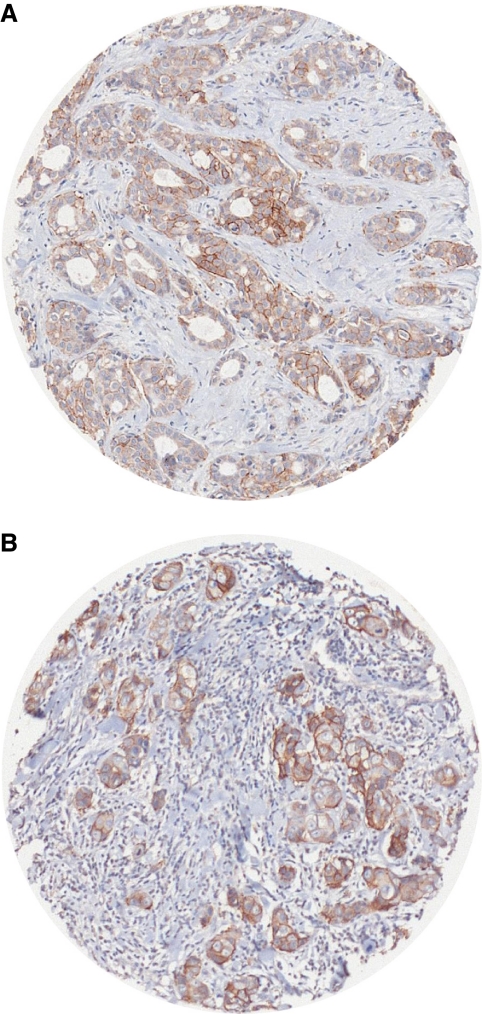
c Kit-positive immunostaining (**A**) in grade 2 breast carcinoma and (**B**) in grade 3 breast carcinomas: ‘spots’ corresponding to tumour cores measuring 0.6 mm in diameter lead to tissue microarray (TMA).

Positive staining was observed in the cell membrane in normal breast and also in tumours, as shown in Figure 1.

The mean quantitative score for c Kit, automatically computed by the image analyser, was significantly higher (*P*=0.0007) in tumours of patients with distant metastasis (*m*=12.2, s.d.=4.3; *n*=181) than in tumours of those lacking distant metastasis (*m*=4.08, s.d.=1.2; *n*=743). Likewise, the mean score was higher (*P*<0.0001) in node-positive tumours (*m*=6.29; s.d.=2.11) than in node-negative tumours (*m*=3.47; s.d.=0.63).

### Prognostic value of c Kit in univariate analysis

**Figure 2:**
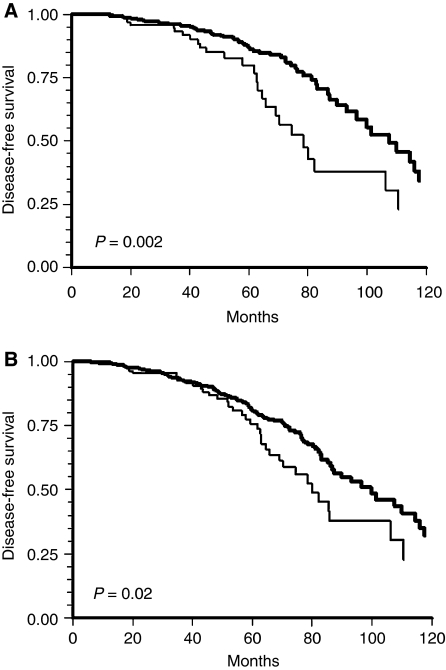
Kaplan–Meier curve showing significant correlation of c Kit densitometry and development of distant metastases (mean follow-up (*P*=0.02 and *P*=0.002 respectively) 79 months) in (**A**) 924 breast carcinoma and (**B**) 586 node-negative tumours.

Comparison of c Kit expression and patients' outcome using log-rank tests showed that c Kit was a significant prognostic indicator in all subgroups (*P*=0.02) and in node-negative patients (*P*=0.002) (Figure 2).

### Multivariate analysis

Multivariate Cox analysis showed that c Kit was an independent prognostic indicator when evaluated with 24 other prognostic indicators categorised as such, using the same quantitative procedure as that in univariate analysis (log-rank test, *P*<0.01), in the whole series of 924 tumours and in the 584 node-negative tumours.

**Figure 3:**
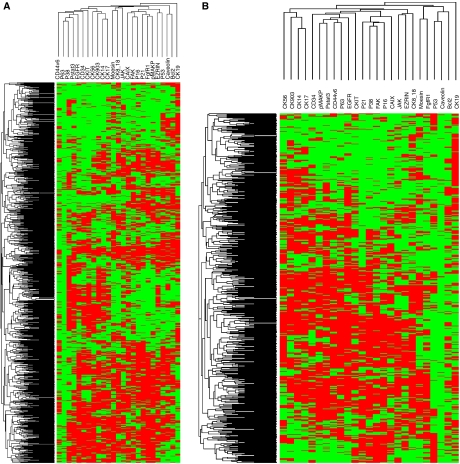
Unsupervised hierarchical clustering of 25 (individually prognostic significant often log-rank tests) markers evaluated by quantitative immunohistochemical (image analysis/densitometry) expression, on tissue microarray (TMA) in (**A**) 924 breast carcinomas and (**B**) 586 node-negative carcinomas.

Hierarchical unsupervised clustering (Figure 3) showed the relationship of c Kit with the other markers, independently of hormone receptor and HER-2 status.

**Figure 4:**
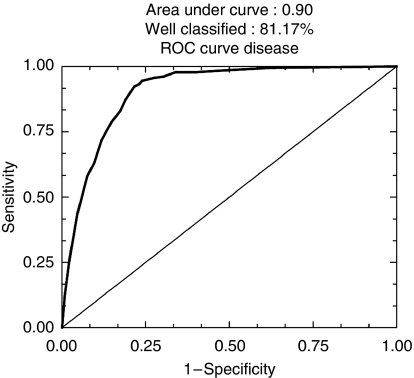
ROC curves after logistic regression of quantitative immunohistochemical expression of 25 prognostic markers in breast carcinomas on tissue microarray (TMA): in whole series (*n*=924) (81.17% of well-classified patients), first step of regression.

**Figure 5:**
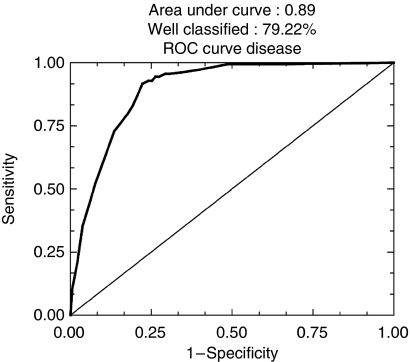
ROC curves after logistic regression of quantitative immunohistochemical expression of 25 prognostic markers in breast carcinomas on tissue microarray (TMA): second step of regression (79.22% of well-classified patients).

**Figure 6:**
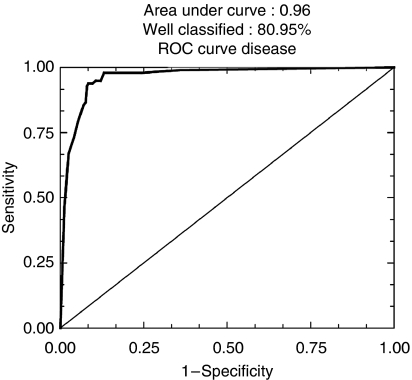
ROC curves after logistic regression of quantitative immunohistochemical expression of 25 prognostic markers in breast carcinomas on tissue microarray (TMA): in node-negative carcinomas (*n*=586) first step (80.95% of well-classified patients).

**Figure 7:**
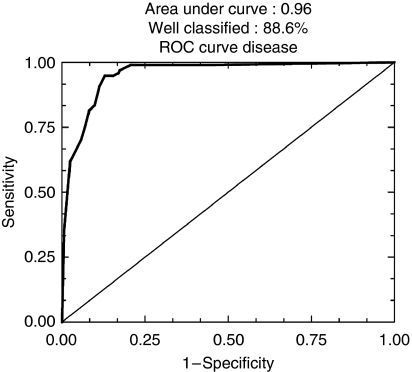
ROC curves after logistic regression of quantitative immunohistochemical expression of 25 prognostic markers in breast carcinomas on tissue microarray (TMA): second step (88.6% of well-classified patients).

To determine the prognostic value, the ranking of c Kit was compared with that of 25 other markers in logistic regression in the series of 924 patients and in the 584 node-negative tumours, independently of ER, PR and HER-2 expression (Tables 2 and 3, Figures 4, 5, 6 and 7).

The first step of logistic regression in the series of 924 tumours showed that 10 out of the 25 markers tested allowed correct classification of 81.17% patients in both categories of good and poor prognosis (sensitivity 78.5%, specificity 92.3%, area under ROC curve 0.906), as shown in Figure 4, and the ranking of c Kit, based on odds ratio and *P*-value of deviance increase (*P*=0.012), was 10 out of 25 (Table 2).

When a second regression step was assessed with the significant markers from the first step, including c Kit, the results showed that a slightly lower percentage of patients (79.22%) were well classified (sensitivity 75.9%, specificity 92.8%, area under ROC curve 0.89) (Figure 5), as compared with the first regression step (81.17%). All 10 markers including c Kit remained highly significant for prognostic prediction of metastasis (*P*-value of deviance increase=0.0411).

Interestingly, when the 584 node-negative tumours were considered, the first-step regression showed that c Kit ranked second among the six-marker signature (Table 3) that correctly classified 80.95% of the patients (sensitivity 80.4%, specificity 83.8%, area under ROC curve 0.960) (Figure 6) in the metastatic or metastasis-free subsets. Moreover, c Kit remained very prognostically significant (*P*<0.0001) along with six others. The ranking of c Kit was 2 out of 25, based on odd ratios and *P*-values of deviance increase (Table 3).

Finally, when a second step of regression was carried out in this node-negative subset with only the six most prognostically significant markers out of 25, patients were well classified in 88.6% (sensitivity 90.3%, specificity 86.5%; area under ROC curve 0.96; Figure 7) and c Kit remained clearly a prognostic predictor and still ranked 2 out of 6.

## Discussion

Breast cancer is a heterogeneous disease, encompassing a number of distinct biological entities that are associated with specific morphological and immunohistochemical features and clinical behaviour (Simpson *et al*, 2005). Despite this morphological heterogeneity, however, patients can practically be classified into three main groups for management and therapy according to: (1) hormone receptor (ER, PR) positivity, (2) presence of Her-2 neu (c-erb B2) amplification or (3) absence of these two characteristics. In the latter group of patients lacking specific targets for hormone and trastuzumab therapy, there is a need to identify new targets for tailored treatments. Genome microarray analysis (Perou *et al*, 2000; Sorlie *et al*, 2003) and expression profiling (Reis-Filho *et al*, 2006) have recently been used to characterise five groups of breast cancers that can also be identified by immunohistochemical screening. In particular, for the triple-negative (ER-PgR-HER-negative) tumours, this deeper molecular insight into tumour characterisation should allow new targets for tailored therapies to be identified. In this regard, the inhibition of c Kit gene expression by imatinib (STI 571, Glivec), which was initially shown to be effective in the treatment of chronic myeloid leukaemia (Kantarjian *et al*, 2002), has more recently been found effective also against c Kit-positive gastrointestinal stroma tumours (GIST) (Miettinen *et al*, 2001), suggesting that other c Kit-positive tumours, in particular breast carcinomas, may respond to imatinib therapy.

In breast cancer, expression of c Kit is reported to be reduced and detected in 1–13% of tumours (Tsuura *et al*, 2002; Nielsen *et al*, 2004; Simon *et al*, 2004; Tsuda *et al*, 2005; Reis-Filho and Tutt, 2008). Our results are close to the upper range of previous reports, with 14.7% of positive tumours in our series. However, some conflicting results have also recently been reported showing a decrease of cKit expression in advanced stage and poor prognosis in breast cancer (Tsutsui *et al*, 2006). The variations in the literature data probably result from the diversity of immunodetection procedures used and also from the lack of results quantification. In this respect, automated quantification of immunoreactions in sections of hundreds of 0.6-micron-thick micro-biopsy cores, using dedicated software to measure extents of staining by densitometry after ‘cropping’ on digitised microscopic images of immunostained TMA, provides a time- and cost-effective, reproducible and accurate means of evaluation, particularly in comparison with approximate and subjective semi-quantitative methods.

Literature data show that c Kit expression in breast cancer is more common in basal-like carcinomas (31%), medullary (19%), grade 3 (24%) and metaplastic (57%) carcinomas (Nielsen *et al*, 2004; Reis-Filho and Tutt, 2008). In our study, c Kit staining was also found to be significantly more highly expressed in more aggressive, node-positive tumours than in node-negative carcinomas (results not shown).

Our results show that c Kit is part of an immunohistochemical signature that permits correct classification of 81.17% patients in the metastatic or metastasis-free categories (mean follow-up 79 months), and 80.95% of node-negative patients. Patients could thus be selected for more aggressive therapy according to evidence from analysis of this immunohistochemical signature at the time of diagnosis, which can be carried out with only a small amount of fixed and paraffin-embedded tissue (7 to 10 4-micron-thick tissue sections) remaining in blocks after microscopic diagnosis and pTN staging. Moreover, this procedure is significantly less time-consuming, as well as cheaper to carry out, than molecular (genome and transcriptome) profiling, so that the results can be available within the same timescale as the pathological report. Thus, standardised quantification of this immunohistochemical signature including cKit (using automated image analysis) could be suitable to examine individual tumours in routine clinicopathological practice, in both node-positive and -negative breast cancers.

Likewise, the prognostic significance of c Kit expression in tumours, in conjunction with the other markers of the signature established by logistic regression, may allow selection of patients for more aggressive therapy, particularly with node-negative tumours. Also, c Kit expressed in the 14.7% of tumours (included in our series) that were positive may serve as a target for specific therapy with imatinib. However, in contrast to GIST, for which imatinib has proved to be an efficient tailored therapy, experience with imatinib therapy in breast cancer is limited. A trial conducted by Modi *et al* (2005) did not establish clinical benefit. However, in that study, no c Kit expression was detected in 8 out of the 11 patients enroled with available tissue. In another pilot study, 9 out of 10 patients enroled with moderate expression of cKit in tumours were partially responsive to imatinib associated with aromatase inhibitors (Chow *et al*, 2008).

In GIST, accumulation of c Kit is usually related to activating mutations. In breast cancer, no mutation has so far been found, though reports referred to very short patient series (*n*=10) (Simon *et al*, 2004). The relationships of c Kit expression and mutations, and patients' response to imatinib-tailored therapy deserve further investigation in conjunction with clinical trials to gain deeper insight into pathways of c Kit regulation and signalling in breast cancer.

In conclusion, our study shows that, as assessed with our high-throughput quantitative immunohistochemical procedure in TMAs from 924 breast cancers, c Kit was expressed in 14.7% of patients and was predictive of patients' outcome, and also in node-negative subsets. Evaluation of c Kit concomitantly with 10 or 6 other prognostic markers by the same method provides a cost-effective procedure permitting the correct classification of 81.17–88.6% of the patients into the metastatic and metastasis-free categories, independent of hormone receptor and HER-2 status, and may be useful in selecting node-negative patients for more aggressive therapy. Finally, for tumours expressing cKit, patients should potentially benefit from tailored therapy with imatinib, in a similar manner to the use of trastuzumab to treat tumours that strongly overexpress HER-2. However, deeper insight into the mechanisms of c Kit downregulation and clinical trials are required to show the relevance of this tailored therapy in breast cancer, as seen with other malignancies such as GIST.

## Figures and Tables

**Table 1:** Sources of antibodies for immunodetection in tissue microarrays

**Antibody**	**Supplier**	**Source**	**Clone**
CD117 (c-Kit)	Dako	Rpab	
E-Cadherin	Zymed	Mmab	4A2C7
CAIX	Abcam	Rpab	
Cytokeratin 903	Dako	Mmab	34BE12
P63	Dako	Mmab	4A4
FYN	Abcam	Mmab	1S
SHARP 2	Abcam	Rpab	
P21Waf1-Cip1	Cell Signaling	Mmab	DCS60
P27 Kip1	Cell Signaling	Rpab	
P38 MAP kinase	Cell Signaling	Rpab	
FAK	Cell Signaling	Rpab	
STAT-1	Cell Signaling	Mmab	9H2
EGFR	Ventana	Mmab	3C6
Phospho-MAPKAPK-2	Cell Signaling	Rmab	(Thr334)
Cytokeratin 19	Dako	Mmab	BA17
Vimentin	Immunotech	Mmab	V9
CD34	Dako	Mmab	QBEnd-10
CD10	Novocastra	Mmab	56C6
STAT-3	Cell Signaling	RMab	Tyr 705 D3A7
Cytokeratin 17	Dako	Mmab	E3
Moesin 1	Biomeda	Mmab	38/87
CD44v6	Novocastra	Mmab	VFF-7
Ezrin(p81,80k,cytovillin)	Neomarkers	Mmab	3C12
FGFR-1 Flg (C-15)	Santa Cruz	Rpab	
P16	Neomarkers	Mmab	Ab7(16PO7)
P53	Dako	Mmab	DO-7
Bcl2	Dako	Mmab	124
CD146	Novocastra	Mmab	N1238
Caveolin 1	Santa Cruz	Rpab	
c-Met	Chemicon/Abcys	Mmab	4AT44
JAK 1	Cell Signaling	Rpab	
Cytokeratins 5-6	Dako	Mmab	D5/16B4

Abbreviations: Mmab=mouse monoclonal antibody; Rpab=rabbit polyclonal antibody.

**Table 2:** Logistic regression of 25 immunohistochemical markers (quantitative score from image analysis) in tissue microarrays of 924 breast carcinomas

	**Marker**	**Odds ratio**	***P*-value of deviance increase**
1	**Moes**	**0.6939**	**0.0000**
2	**P53**	**0.4091**	**0.0000**
3	**pMAPK**	**1.4654**	**0.0000**
4	**P38**	**07979**	**0.0026**
5	**CaIX**	**1.1941**	**0.0041**
6	**CD44v6**	**1.1866**	**0.0071**
7	**CK903**	**0.8573**	**0.0191**
8	**P21**	**0.8577**	**0.0249**
9	**CAVEOL**	**0.7986**	**0.0322**
10	**c kit**	**0.8816**	**0.0411**
11	CK5-6	1.1262	0.0654
12	JAK	1.1110	0.0711
13	K8-18	0.8925	0.0864
14	P16	0.9078	0.1292
15	EGFR	1.1024	0.1392
16	K14	1.1062	0.1471
17	CD34	0.9292	0.2282
18	P63	1.0754	0.3291
19	FAK	0.9400	0.3666
20	PSTAT3	0.9471	0.4750
21	Bcl2	1.0452	0.4968
22	CK19	1.0413	0.5140
23	Ezrin	0.9648	0.5737
24	FgFR1	0.9792	0.7484
25	CK17	0.9999	0.9982

Bold digits signify markers found to be prognostically significant.

**Table 3:** Logistic regression of 25 immunohistochemical markers (quantitative score from image analysis) in tissue microarrays of 586 node-negative breast carcinomas

	**Markers**	**Odds ratio**	***P*-value of deviance increase**
1	**P53**	**0.3290**	**<0.0000**
2	**c kit**	**0.6190**	**<0.0000**
3	**P21**	**0.6190**	**0.0001**
4	**CK903**	**0.7331**	**0.0082**
5	**Moes**	**0.7369**	**0.0204**
6	**CK17**	**1.3529**	**0.0217**
7	EGFR	1.2385	0.0553
8	CD44V6	1.3099	0.0609
9	Bcl2	1.2337	0.0679
10	P38	1.2730	0.0793
11	pMAPK	1.2851	0.0889
12	JAK	1.1817	0.1144
13	FgFR1	0.8399	0.1194
14	P16	1.2008	0.1495
15	CaIX	1.1726	0.1766
16	CK8-18	1.1561	0.2086
17	FAK	0.8568	0.2249
18	Ezrin	1.0852	0.4662
19	P63	1.0564	0.6560
20	CK14	1.0375	0.7727
21	PSTAT3	1.0262	0.8695
22	CK19	1.0157	0.8876
23	KER56	1.0121	0.9157
24	CAVEOL	1.0286	0.9223
25	CD34	0.9908	0.9341

Bold digits signify markers found to be prognostically significant.

## References

[bib1] Altman DG, Lausen B, Sauerbrei W, Schumacher M (1994) Dangers of using ‘optimal’ cutpoints in the evaluation of prognostic factors. J Natl Cancer Inst 86: 829–835 8182763 10.1093/jnci/86.11.829

[bib2] Charpin C, Dales JP, Garcia S, Carpentier S, Djemli A, Andrac L, Lavaut MN, Allasia C, Bonnier P (2004) Tumor neoangiogenesis by CD31 and CD105 expression evaluation in breast carcinoma tissue microarrays. Clin Cancer Res 10: 5815–5819 15355911 10.1158/1078-0432.CCR-04-0021

[bib3] Charpin C, Garcia S, Bonnier P, Martini F, Andrac L, Choux R, Lavaut MN, Allasia C (1998a) Reduced E-cadherin immunohistochemical expression in node-negative breast carcinomas correlates with 10-year survival. Am J Clin Pathol 109: 431–438 9535397 10.1093/ajcp/109.4.431

[bib4] Charpin C, Garcia S, Bonnier P, Martini F, Andrac L, Horschowski N, Lavaut MN, Allasia C (1998b) Prognostic significance of Nm23/NDPK expression in breast carcinoma, assessed on 10-year follow-up by automated and quantitative immunocytochemical assays. J Pathol 184: 401–407 9664906 10.1002/(SICI)1096-9896(199804)184:4<401::AID-PATH1220>3.0.CO;2-U

[bib5] Charpin C, Garcia S, Bonnier P, Martini F, Andrac L, Horschowski N, Lavaut MN, Allasia C (1998c) bcl-2 automated and quantitative immunocytochemical assays in breast carcinomas: correlation with 10-year follow-up. J Clin Oncol 16: 2025–2031 9626199 10.1200/JCO.1998.16.6.2025

[bib6] Charpin C, Garcia S, Bouvier C, Devictor B, Andrac L, Choux R, Lavaut MN, Allasia C (1997a) Automated and quantitative immunocytochemical assays of CD44v6 in breast carcinomas. Hum Pathol 28: 289–296 9042792 10.1016/s0046-8177(97)90126-x

[bib7] Charpin C, Garcia S, Bouvier C, Martini F, Andrac L, Bonnier P, Lavaut MN, Allasia C (1997b) CD31/PECAM automated and quantitative immunocytochemical assays in breast carcinomas: correlation with patient follow-up. Am J Clin Pathol 107: 534–541 9128265 10.1093/ajcp/107.5.534

[bib8] Charpin C, Vielh P, Duffaud F, Devictor B, Andrac L, Lavaut MN, Allasia C, Horschowski N, Piana L (1994) Quantitative immunocytochemical assays of P-glycoprotein in breast carcinomas: correlation to messenger RNA expression and to immunohistochemical prognostic indicators. J Natl Cancer Inst 86: 1539–1545 7932810 10.1093/jnci/86.20.1539

[bib9] Chow LW, Yip AY, Loo WT, Toi M (2008) Evaluation of neoadjuvant inhibition of aromatase activity and signal transduction in breast cancer. Cancer Lett 262: 232–238 18248884 10.1016/j.canlet.2007.12.003

[bib10] Garcia S, Dales JP, Charafe-Jauffret E, Carpentier-Meunier S, Andrac-Meyer L, Jacquemier J, Andonian C, Lavaut MN, Allasia C, Bonnier P, Charpin C (2007a) Overexpression of c-Met and of the transducers PI3K, FAK and JAK in breast carcinomas correlates with shorter survival and neoangiogenesis. Int J Oncol 31: 49–58 17549404

[bib11] Garcia S, Dales JP, Charafe-Jauffret E, Carpentier-Meunier S, Andrac-Meyer L, Jacquemier J, Andonian C, Lavaut MN, Allasia C, Bonnier P, Charpin C (2007b) Poor prognosis in breast carcinomas correlates with increased expression of targetable CD146 and c-Met and with proteomic basal-like phenotype. Hum Pathol 38: 830–841 17316758 10.1016/j.humpath.2006.11.015

[bib12] Kantarjian H, Sawyers C, Hochhaus A, Guilhot F, Schiffer C, Gambacorti-Passerini C, Niederwieser D, Resta D, Capdeville R, Zoellner U, Talpaz M, Druker B, Goldman J, O'Brien SG, Russell N, Fischer T, Ottmann O, Cony-Makhoul P, Facon T, Stone R, Miller C, Tallman M, Brown R, Schuster M, Loughran T, Gratwohl A, Mandelli F, Saglio G, Lazzarino M, Russo D, Baccarani M, Morra E (2002) Hematologic and cytogenetic responses to imatinib mesylate in chronic myelogenous leukemia. N Engl J Med 346: 645–652 11870241 10.1056/NEJMoa011573

[bib13] Matsuda R, Takahashi T, Nakamura S, Sekido Y, Nishida K, Seto M, Seito T, Sugiura T, Ariyoshi Y, Takahashi T, Ueda R (1993) Expression of the c-kit protein in human solid tumors and in corresponding fetal and adult normal tissues. Am J Pathol 142: 339–346 7678721 PMC1886849

[bib14] Miettinen M, Furlong M, Sarlomo-Rikala M, Burke A, Sobin LH, Lasota J (2001) Gastrointestinal stromal tumors, intramural leiomyomas, and leiomyosarcomas in the rectum and anus: a clinicopathologic, immunohistochemical, and molecular genetic study of 144 cases. Am J Surg Pathol 25: 1121–1133 11688571 10.1097/00000478-200109000-00002

[bib15] Miettinen M, Lasota J (2005) KIT (CD117): a review on expression in normal and neoplastic tissues, and mutations and their clinicopathologic correlation. Appl Immunohistochem Mol Morphol 13: 205–220 16082245 10.1097/01.pai.0000173054.83414.22

[bib16] Modi S, Seidman AD, Dickler M, Moasser M, D'Andrea G, Moynahan ME, Menell J, Panageas KS, Tan LK, Norton L, Hudis CA (2005) A phase II trial of imatinib mesylate monotherapy in patients with metastatic breast cancer. Breast Cancer Res Treat 90: 157–163 15803362 10.1007/s10549-004-3974-0

[bib17] Nielsen TO, Hsu FD, Jensen K, Cheang M, Karaca G, Hu Z, Hernandez-Boussard T, Livasy C, Cowan D, Dressler L, Akslen LA, Ragaz J, Gown AM, Gilks CB, van de Rijn M, Perou CM (2004) Immunohistochemical and clinical characterization of the basal-like subtype of invasive breast carcinoma. Clin Cancer Res 10: 5367–5374 15328174 10.1158/1078-0432.CCR-04-0220

[bib18] Perou CM, Sorlie T, Eisen MB, van de Rijn M, Jeffrey SS, Rees CA, Pollack JR, Ross DT, Johnsen H, Akslen LA, Fluge O, Pergamenschikov A, Williams C, Zhu SX, Lonning PE, Borresen-Dale AL, Brown PO, Botstein D (2000) Molecular portraits of human breast tumours. Nature 406: 747–752 10963602 10.1038/35021093

[bib19] Reis-Filho JS, Tutt AN (2008) Triple negative tumours: a critical review. Histopathology 52: 108–118 18171422 10.1111/j.1365-2559.2007.02889.x

[bib20] Reis-Filho JS, Westbury C, Pierga JY (2006) The impact of expression profiling on prognostic and predictive testing in breast cancer. J Clin Pathol 59: 225–231 16505270 10.1136/jcp.2005.028324 PMC1860331

[bib21] Simon R, Panussis S, Maurer R, Spichtin H, Glatz K, Tapia C, Mirlacher M, Rufle A, Torhorst J, Sauter G (2004) KIT (CD117)-positive breast cancers are infrequent and lack KIT gene mutations. Clin Cancer Res 10: 178–183 14734467 10.1158/1078-0432.ccr-0597-3

[bib22] Simpson PT, Reis-Filho JS, Gale T, Lakhani SR (2005) Molecular evolution of breast cancer. J Pathol 205: 248–254 15641021 10.1002/path.1691

[bib23] Sorlie T, Tibshirani R, Parker J, Hastie T, Marron JS, Nobel A, Deng S, Johnsen H, Pesich R, Geisler S, Demeter J, Perou CM, Lonning PE, Brown PO, Borresen-Dale AL, Botstein D (2003) Repeated observation of breast tumor subtypes in independent gene expression data sets. Proc Natl Acad Sci USA 100: 8418–8423 12829800 10.1073/pnas.0932692100 PMC166244

[bib24] Tsuda H, Morita D, Kimura M, Shinto E, Ohtsuka Y, Matsubara O, Inazawa J, Tamaki K, Mochizuki H, Tamai S, Hiraide H (2005) Correlation of KIT and EGFR overexpression with invasive ductal breast carcinoma of the solid-tubular subtype, nuclear grade 3, and mesenchymal or myoepithelial differentiation. Cancer Sci 96: 48–53 15649255 10.1111/j.1349-7006.2005.00009.x PMC11160055

[bib25] Tsutsui S, Yasuda K, Suzuki K, Takeuchi H, Nishizaki T, Higashi H, Era S (2006) A loss of c-kit expression is associated with an advanced stage and poor prognosis in breast cancer. Br J Cancer 94: 1874–1878 16721362 10.1038/sj.bjc.6603183 PMC2361342

[bib26] Tsuura Y, Suzuki T, Honma K, Sano M (2002) Expression of c-kit protein in proliferative lesions of human breast: sexual difference and close association with phosphotyrosine status. J Cancer Res Clin Oncol 128: 239–246 12029439 10.1007/s00432-002-0329-2 PMC12164464

